# An assessment of sensory sensitivity, including certain anhedonia dimensions, in patients with schizophrenia using transcutaneous electrical nerve stimulation

**DOI:** 10.1371/journal.pone.0344587

**Published:** 2026-03-06

**Authors:** Joanna Witkoś, Agnieszka Fusińska-Korpik, Krzysztof Walczewski

**Affiliations:** 1 Collegium Medicum, Department of Health Science, Andrzej Frycz Modrzewski Krakow University, Kraków, Poland; 2 Józef Babiński University Hospital in Krakow, Psychiatric Ward, Kraków, Poland; University of California Davis, UNITED STATES OF AMERICA

## Abstract

This study addresses a largely unexplored area in schizophrenia: the relationship between affective functioning and sensory perception assessed through objective, quantifiable methods. To the best of the authors’ knowledge, this is the first study to assess the electrical sensory threshold in individuals with schizophrenia using transcutaneous electrical nerve stimulation (TENS). It was hypothesized that patients with schizophrenia require higher current intensity to reach the sensory threshold and that an association exists between anhedonia and the subjective evaluation of the electrical stimulus. The study was based on an objective assessment of the sensory threshold using electrical stimulation. A gradually increasing electrical pulse was applied until the sensory threshold, defined as the minimum tingling sensation perceived by the participant, was reached. The study included 108 individuals diagnosed with schizophrenia and 59 participants in the control group. Various dimensions of anhedonia were assessed using the Dimensional Anhedonia Rating Scale (DARS). Quality of life and its domains were assessed using the WHOQOL-BREF. Participants reporting higher levels of sensory anhedonia rated the electrical stimulus as less pleasant. A negative correlation was observed between the pleasantness of the stimulus and both patient age and treatment duration. Furthermore, individuals whose clinical improvement was rated by the physician as more pronounced than at baseline exhibited greater sensitivity to the electrical stimulus. These findings suggest that anhedonia should be considered not only as a core symptom of schizophrenia, but also as a factor influencing patients’ adaptation in multiple domains, including sensory processing. Addressing anhedonia may therefore be a relevant target for both preventive strategies and rehabilitation programs.

## Introduction

Sensory processing sensitivity (SPS) is an evolutionarily conserved and biologically grounded trait that reflects stable individual differences in the perception and reactivity to environmental stimuli. These stimuli may be either positive or negative, and individuals vary not only in their detection thresholds but also in their affective and behavioral responses to sensory stimulation [1,2]. A person’s sensory profile encompasses both neurological thresholds and the corresponding behavioral responses. Neurological thresholds determine the intensity a stimulus must reach to be noticed or to evoke a reaction. Individuals with low thresholds may perceive even weak stimuli as intense or overwhelming, whereas those with high thresholds may not detect the same stimuli at all. Behavioral responses reflect how a person acts under sensory input, they may align with neurological sensitivity or compensate for it [3–5]. SPS is not a disorder in itself but may lead to dysfunction when environmental demands exceed an individual’s adaptive capacity.

Sensory processing disorders (SPDs) refer to clinically significant difficulties in the detection, modulation, and integration of sensory input across modalities. These disturbances often lead to disorganized or contextually inappropriate behavioral responses [6,7]. Although SPDs have been predominantly investigated in pediatric populations, their relevance in adult mental health is increasingly recognized, particularly in severe psychiatric disorders. SPDs may manifest as either hypersensitivity or hyposensitivity. Hyposensitivity may involve reduced sensory registration or, conversely, increased active seeking of sensory input. In contrast, hypersensitivity may be characterized by impaired sensory filtering despite perceived unpleasantness, or by active avoidance strategies aimed at reducing exposure to aversive stimuli [8,9]. There remains ongoing debate as to whether SPD constitutes a distinct clinical entity or represents a transdiagnostic feature shared across psychiatric conditions [10]. Dunn’s Sensory Processing Framework conceptualizes these patterns along two dimensions: neurological threshold (low–high) and behavioral strategy (active–passive), enabling a structured characterization of sensory processing styles such as seeking, avoidance, and under-registration [4,5].

Schizophrenia is a chronic and highly disabling neuropsychiatric disorder affecting approximately 1% of the global population. Beyond its core clinical features, including hallucinations, delusions, and cognitive impairment, increasing attention has been directed toward sensory processing disturbances as a potentially fundamental component of the disorder [3]. Individuals with schizophrenia may exhibit both heightened sensitivity to sensory input, often experienced as overwhelming, and reduced sensitivity characterized by delayed or attenuated responses. Neuroimaging findings suggest structural and functional abnormalities within sensory cortical regions, alongside disrupted connectivity between sensory and higher-order associative networks [11]. Deficits in multisensory integration, particularly involving proprioceptive and tactile modalities, have been proposed as contributing factors in the development of psychotic symptoms, including hallucinations [12]. Furthermore, impairments in sensory gating, the capacity to suppress irrelevant sensory information, are commonly observed. Consequently, incoming stimuli may be processed as equally salient, potentially leading to perceptual overload and behavioral withdrawal. The coexistence of hyper- and hyposensitive responses, varying across contexts and stimulus characteristics, underscores the heterogeneity and complexity of sensory processing alterations in schizophrenia. Due to the complexity of sensory processing, findings across clinical populations, including individuals with schizophrenia, remain inconsistent and are often based on heterogeneous methodological approaches. Importantly, only a limited number of studies have employed objective assessment methods [13], with the majority relying predominantly on self-report measures. The integration of objective measurement techniques may therefore provide more precise insights into the mechanisms and characteristics of sensory processing disturbances in psychiatric populations [3,14]. Within this framework, non-invasive transcutaneous electrical nerve stimulation (TENS) represents a promising and objective approach for assessing sensory thresholds in schizophrenia. The use of a quantifiable electrical stimulus offers a standardized method that may complement subjective reports while enhancing measurement reliability and reproducibility. Objective evaluation of sensory sensitivity may contribute to a more refined understanding of individual differences in sensory processing and their potential relationship with symptom profiles. Moreover, such approaches may help reduce methodological variability, thereby facilitating more consistent comparisons across studies and clinical populations.

Anhedonia, first described by Ribot in 1896 [15], is considered a core negative symptom of schizophrenia [16]. Sensory anhedonia, a distinct subtype, refers to a reduced capacity to experience pleasure from sensory stimuli, including visual, auditory, gustatory, and tactile input [17]. Despite its clinical relevance, sensory anhedonia has been only rarely examined using objective assessment methods. Most existing studies rely on self-report questionnaires, which are inherently constrained by subjective bias and methodological limitations [18]. Given the critical role of sensory experiences in emotional regulation and adaptive functioning, investigating anhedonia in relation to objectively measured sensory thresholds may provide additional insights into the mechanisms underlying schizophrenia. The present study aimed to assess sensory sensitivity thresholds to electrical stimulation in individuals diagnosed with schizophrenia using non-invasive TENS. Additionally, we sought to examine the sensory dimensions of anhedonia and their potential determinants. To the best of the authors’ knowledge, this study represents the first application of TENS for the objective assessment of sensory thresholds in this clinical population.

## Materials and methods

### Participants

The study included 108 individuals diagnosed with schizophrenia: 30 women (27.77%) and 78 (72.22%) men with a mean age of 39.90 ± 8.11 years (mean ± standard deviation). The control group consisted of 59 healthy participants with a mean age of 31.53 ± 7.72 years. The number of women was identical in both groups (n = 30), whereas the proportion of men was higher in the schizophrenia group (Table 1). The mean duration of treatment among patients with schizophrenia was 9.47 ± 5.78 years. The mean treatment duration was longer in women (12.83 ± 5.69 years) than in men (8.18 ± 5.31 years). All patients were hospitalised in general psychiatric inpatient wards of the Dr. Józef Babiński Clinical Hospital in Kraków, where the study was conducted. Participant recruitment was carried out between July 20, 2022, and December 10, 2023. Written informed consent was obtained from all participants included in the study.

**Table 1:** Sex distribution in the schizophrenia and control group.

	Schizophrenia group		Control group		Total	
	N	%	N	%	N	%
Female	30	27.78	30	50.85	60	35.93
Male	78	72.22	29	49.15	107	64.07
Total	108	100.00	59	100.00	167	100.00

*chi*^*2*^(1) = 8.82; *p* =.003, Cramer’s V = 0.23

### Procedure

Before the study, participants completed an anonymous questionnaire including items on demographic characteristics, psychiatric diagnosis, number of hospitalisations, and current use of psychotropic medications. Symptom severity was rated by a psychiatrist using the Clinical Global Impressions – Improvement (CGI-I) scale [19]. Anhedonia was evaluated with the Dimensional Anhedonia Rating Scale (DARS) [20], using the validated Polish adaptation [21]. The DARS assesses motivation and hedonic capacity across both physical and psychosocial domains. Depressive symptoms were measured using the Beck Depression Inventory (BDI), while quality of life was assessed with the WHOQOL-BREF questionnaire, covering physical, psychological, social, and environmental domains.

Sensory threshold assessment was conducted using an electrotherapeutic stimulation device (BTL-5818SLM Combi; part no. P5818.129; BTL Medical Technologies s.r.o./BTL Industries, Prague, Czech Republic) with a current resolution of 0.1 mA. A gradually increasing electrical current was applied as the sensory stimulus until the sensory threshold was reached, defined as the minimum tingling sensation perceived by the participant. TENS was applied with the following parameters: 100 Hz frequency, 100 µs pulse duration, and a biphasic waveform. Surface electrodes were placed on the forearm of the participant’s dominant hand [22].

The inclusion criteria were a confirmed diagnosis of schizophrenia established by a psychiatrist. Eligibility was determined by the attending physician in consultation with a clinical psychologist. The exclusion criteria included the presence of an organic etiology of the mental disorder and partial or complete legal incapacitation.

Capacity to consent was assessed by the treating psychiatrist based on the participant’s current mental status, clinical presentation, and ability to understand the nature and procedures of the study. Only individuals considered capable of providing consent were included. All participants provided written informed consent after receiving a full explanation of the study procedures. This study was conducted in accordance with the Declaration of Helsinki, and approved by the Bioethical Committee of Andrzej Frycz Modrzewski Krakow University (permission number KBKA/33/O/2022).

### Statistical analyses

Group differences between participants with schizophrenia and control subjects were examined using a generalized linear model with an identity link function, adjusting for age and sex. Because the majority of variables showed substantial deviations from normality, robust standard errors (Huber–White sandwich estimator) were applied to ensure valid statistical inference without relying on distributional assumptions [23]. Results are presented with 95% confidence intervals. Statistical analyses were performed using IBM SPSS Statistics (PS IMAGO), version 29 (IBM Corp., Armonk, NY, USA).

## Results

Statistical analysis revealed highly significant differences (p < 0.001) between the control and schizophrenia groups in the first sensory threshold measurement, as well as across all four anhedonia domains (Table 2). A significant between-group difference was also observed for the mean current intensity calculated from the two measurements required to determine the sensory threshold (p = 0.001). There were no statistically significant differences in the hedonic ratings of the electrical stimulus between the groups, with the stimulus perceived as neutral by the majority of participants. Notably, a higher proportion of control participants rated the stimulus as unpleasant (15.25%) compared with individuals with schizophrenia (6.48%) (Table 3).

**Table 2:** Descriptive statistics and results of the Generalized Linear Models for the difference between groups (age and sex controlled for).

	Mean		Q1		Median		Q3		B	Lower 95%CI	Higher 95%CI	Wald chi^2^	p
	(C)	S	C	S	C	S	C	S					
SENSORY THRESHOLD MEASUREMENTS													
First measurement	7.09 ± 2.12	9.15 ± 3.72	5,50	6,30	6,50	8,90	8,70	12,75	−1.86	−2.86	−0.86	13.37	<.001
Second measurement	6.80 ± 2.14	8.51 ± 3.70	5,10	5,33	6,40	9,00	8,50	11,00	−1.50	−2.53	−0.47	8.11	.004
Average of first and second measurements	6.94 ± 2.13	8.83 ± 3.68	5,40	5,70	6,50	9,05	8,60	11,69	−1.68	−2.69	−0.67	10.70	.001
DARS													
sensory experiences	21.97 ± 1.66	20.84 ± 4.58	21,00	18,00	22,00	21,00	23,00	25,00	1.10	−0.15	2.35	2.99	.084
hobbies	17.42 ± 1.52	17.66 ± 3.66	17,00	16,00	17,00	19,00	18,00	20,00	0.06	−1.00	1.13	0.01	.906
food/drinks	17.39 ± 1.63	16.00 ± 3.89	16,00	13,25	17,00	17,00	18,00	20,00	1.25	0.25	2.25	6.01	.014
social activities	16.78 ± 1.87	16.06 ± 3.66	15,00	13,00	16,00	16,00	18,00	20,00	0.09	−0.95	1.14	0.03	.863
Total	73.56 ± 3.88	70.56 ± 11.71	71,00	63,25	74,00	73,00	76,00	80,50	2.51	−0.40	5.42	2.86	.091
DOMAINS													
somatic	71.19 ± 11.81	50.13 ± 17.50	60,71	35,71	71,43	50,00	78,57	60,71	22.59	17.33	27.84	70.95	<.001
psychological	69.92 ± 12.48	51.00 ± 19.90	62,50	34,38	75,00	54,17	79,17	65,63	19.72	14.16	25.28	48.32	<.001
social	75.85 ± 17.83	47.15 ± 20.95	66,67	33,33	75,00	50,00	91,67	58,33	29.63	23.41	35.86	87.09	<.001
environmental	71.77 ± 10.89	54.05 ± 16.87	62,50	43,75	71,88	56,25	81,25	65,63	17.40	12.35	22.45	45.63	<.001

**Table 3:** Distribution of hedonic responses to the electrical stimulus in the study groups.

		Schizofrenia group		Control group		Total	
		N	%	N	%	N	%
Hedonic rating	Neutral	84	77.78	42	71.19	126	75.45
	Pleasant	17	15.74	8	13.56	25	14.97
	Unpleasant	7	6.48	9	15.25	16	9.58
	Total	108	100.00	59	100.00	167	100.00

*chi*^*2*^(2) = 3.41; *p* =.182, Cramer’s V = 0.18

Notably, a significant negative correlation (p < 0.05) was observed between DARS sensory experience scores and the hedonic appraisal of the electrical stimulus, with greater sensory anhedonia associated with less pleasant stimulus perception (Table 4). Treatment duration was also negatively correlated with hedonic stimulus evaluation, indicating that longer treatment was associated with more negative stimulus appraisal. A significant negative association (p < 0.05) was further identified between overall clinical improvement assessed using the CGI-I scale and both sensory threshold measurements. Participants demonstrating greater clinical improvement exhibited lower sensory thresholds, reflecting increased sensitivity to the electrical stimulus (Table 4). Most anhedonia indices, including the total DARS score, were negatively correlated with both treatment duration and age. Older participants and those with longer treatment histories demonstrated reduced hedonic capacity. In contrast, psychological quality of life was positively correlated with overall hedonic capacity. Similarly, pleasure derived from hobbies/interests was positively associated with quality of life in the interpersonal relationships domain. Participants reporting better psychological well-being demonstrated greater overall hedonic functioning (Table 4) (Table 5).

**Table 4:** Partial correlations between sensory thresholds and clinical variables in the clinical group, controlling for age and sex.

	Treatment time [years]	Percption of stimulus	First sensory threshold measurement	Second sensory threshold measurement	Average of first and second sensory threshold measurement	DARS sensory experiences	DARS hobbies	DARS foods/drinks	DARS social activities	DARS TOTAL	Domain somatic	Domain psychological	Domain social	Domain environmental	CGIS
Perception of stimulus	-,39**														
First sensory threshold measurement	,04	-,04													
Second sensory threshold measurement	-,02	,01	,97**												
Average of first and second sensory threshold measurement	,01	-,02	,99**	,99**											
DARS sensory experiences	-,07	-,19	,08	,07	,07										
DARS hobbies	-,13	-,02	,01	-,04	-,01	,33**									
DARS foods/drinks	-,11	,11	,05	,14	,10	,42**	,07								
DARS social activities	-,07	-,05	,01	,05	,03	,56**	,12	,60**							
DARS TOTAL	-,14	-,06	,06	,08	,07	,83**	,54**	,72**	,77**						
Domain somatic	-,05	,18	,10	,08	,09	,08	,20*	,01	,00	,10					
Domain psychologica	-,14	,13	,10	,09	,09	,21*	,20*	,09	,19	,24*	,72**				
Domain social	-,12	,06	,08	,04	,06	,14	,28**	-,05	,10	,16	,58**	,61**			
Domain environmental	-,12	,01	,07	,03	,05	,03	,19	-,06	,10	,09	,48**	,49**	,62**		
CGIS	,04	-,05	,07	,06	,06	,10	,06	,05	,05	,09	,09	,10	,10	,07	
CGII	,00	,10	-,20*	-,22*	-,21*	-,01	,00	,03	-,04	-,01	,16	,15	,07	,11	,04

*: p < 0,05; **: p < 0,01

**Table 5:** Partial correlations between sensory thresholds and clinical variables in the control group, controlling for age and sex.

	Percption of stimulus	First sensory threshold measurement	Second sensory threshold measurement	Average of first and second sensory threshold measurement	DARS sensory experiences	DARS hobbies	DARS foods/drinks	DARS social activities	DARS TOTAL	Domain somatic	Domain psychological	Domain social
First sensory threshold measurement	-,09											
Second sensory threshold measurement	-,08	,99**										
Average of first and second sensory threshold measurement	-,08	1,00**	1,00**									
DARS sensory experiences	-,21	-,05	-,06	-,05								
DARS hobbies	,06	,12	,12	,12	-,10							
DARS foods/drinks	-,10	,01	,01	,01	,02	,23						
DARS social activities	-,28*	,02	,00	,01	,46**	-,12	,20					
DARS TOTAL	-,24	,04	,03	,03	,60**	,39**	,61**	,70**				
Domain somatic	,17	-,07	-,05	-,06	-,09	,07	,13	,12	,10			
Domain psychological	,23	-,09	-,05	-,07	-,24	,27*	,28*	-,09	,09			
Domain social	,22	-,17	-,15	-,16	-,04	,15	,01	-,23	-,06	,30*	,47**	
Domain environmental	,17	-,09	-,07	-,08	,02	-,07	,05	,04	,02	,90**	,35**	,26*

*: p < 0,05; **: p < 0,01

## Discussion

The ability to detect, process, and respond to environmental stimuli reflects stable individual differences in sensory functioning, often conceptualized as a sensory profile. Efficient central processing and modulation of sensory input are essential for adaptive behaviour and effective interaction with the environment. Impaired sensory processing has been proposed as a clinically relevant feature of schizophrenia and may contribute to the emergence of psychotic symptoms and cognitive dysfunction [24].

The present study was based on an objective method of measuring sensory thresholds using electric current. Accordingly, the findings provide novel insights and contribute to the existing body of knowledge on sensory perception in patients with schizophrenia. Specifically, our data indicate that patients with schizophrenia exhibit elevated sensory thresholds, requiring higher current intensities than healthy individuals. While no significant differences were found in hedonic ratings of the stimulus, individuals with schizophrenia were less likely to describe it as unpleasant, suggesting reduced affective sensitivity to discomfort. Notably, longer treatment duration correlated with decreased stimulus pleasantness, indicating potential changes in sensory responsiveness along the illness-recovery trajectory.

Age and treatment duration were negatively associated with anhedonia scores, indicating reduced hedonic capacity in older individuals and those undergoing prolonged treatment. In contrast, better psychological well-being and satisfaction with interpersonal relationships were positively correlated with hedonic capacity, particularly within the domain of hobbies and interests. Reduced pleasantness ratings of the electrical stimulus were observed in participants with higher sensory anhedonia scores, suggesting an interaction between sensory processing and negative symptomatology [25]. Additionally, greater clinician-rated improvement was associated with lower sensory thresholds, indicating increased sensory responsiveness in patients with better treatment outcomes. These findings suggest that sensory reactivity may be linked to clinical improvement, although the underlying mechanisms require further investigation.

Individuals with schizophrenia frequently exhibit atypical neural responses to sensory stimuli and increased sensory avoidance. A meta-analysis of 33 studies employing the Adolescent/Adult Sensory Profile demonstrated consistent patterns of sensory processing disruptions across psychiatric diagnoses, supporting a transdiagnostic interpretation of these phenomena [26]. In schizophrenia, such alterations have been described as reduced tactile sensitivity, impaired multisensory integration [12], and diminished pain responsiveness [27]. Empirical studies further indicate the co-occurrence of seemingly opposing sensory patterns. Brown et al. [3] reported increased sensation avoidance and low registration, alongside reduced sensation seeking, in both schizophrenia and bipolar disorder groups relative to controls. Similarly, Zhou et al. [11] confirmed the simultaneous presence of hypersensitivity and hyposensitivity in adolescents with schizophrenia. Notably, atypical sensory patterns were positively associated with schizotypal traits across diagnostic groups, reinforcing dimensional models of sensory processing abnormalities that extend beyond categorical diagnoses. Converging evidence supports the clinical and mechanistic relevance of sensory processing disturbances in schizophrenia. Sensory modulation interventions have been shown to reduce stress, alleviate symptom burden, and improve occupational functioning [28]. Experimental studies further demonstrate that disruptions in multisensory temporal precision are associated with hallucination severity, highlighting the importance of intact sensory integration for coherent perception [29].

Neurocomputational accounts provide complementary explanations. Hallucination severity has been linked to heightened sensory sensitivity and shorter illness duration, suggesting dynamic alterations in perceptual inference processes [30]. These findings align with predictive coding models proposing dysregulated integration of sensory evidence and prior expectations. Neuroimaging studies additionally indicate altered activation patterns, characterized by reduced engagement of early sensory regions alongside increased recruitment of higher-order cognitive areas, potentially reflecting compensatory mechanisms [31].

Although the course of recurrent psychotic disorders is shaped by multiple factors [32], negative symptoms remain a defining component of chronic schizophrenia [33]. Anhedonia, conceptualised as a diminished capacity to experience pleasure, is widely recognised as a core element of negative symptomatology [34–41]. Empirical evidence indicates that hedonic deficits are closely linked not only to negative symptoms but also to cognitive disorganization [38]. While abnormalities in reward processing are considered a likely underlying mechanism, anhedonia in schizophrenia appears to differ qualitatively from its expression in other psychiatric disorders [39]. It has been proposed that reduced self-reported valuation of past and anticipated pleasures may reflect impairments in higher-order cognitive processes rather than solely primary reward dysfunction [40]. Consistent with these accounts, our findings indicate that older patients and those with longer treatment histories exhibited significantly reduced hedonic capacity. This pattern supports the view that anhedonia represents a central feature of chronic psychotic illness and may partly relate to illness-associated cognitive decline [41].

This study identified significant associations between hedonic capacity and selected domains of quality of life, particularly the psychological and social dimensions. Patients with a greater ability to experience pleasure reported a stronger sense of meaning and subjective value of life. Although no significant relationship was observed between anhedonia and physical quality of life, the present findings are consistent with previous reports indicating that individuals with schizophrenia tend to exhibit reduced quality of life across multiple domains [42]. Collectively, these results underscore the clinical relevance of anhedonia not only as a core symptom of schizophrenia but also as a factor influencing functional adaptation, with potential implications for preventive and rehabilitative interventions.

The obtained results suggest that higher sensory reactivity may be associated with a shorter duration of treatment. Although direct empirical evidence supporting this interpretation remains limited, prior studies have indicated that sensory processing characteristics may relate to treatment responsiveness. This association may reflect neurobiological mechanisms influencing pharmacological response, particularly those involving sensory processing and central nervous system arousal regulation [16,43]. Moreover, sensory reactivity may act as a moderating factor shaping individual responses to psychosocial interventions, potentially through interactions with personality-related variables [44]. Within this framework, sensory reactivity may represent a clinically relevant dimension of interindividual variability that warrants consideration in treatment planning and personalization. Further research is needed to clarify the mechanisms underlying these associations and to determine whether sensory reactivity may serve as a meaningful predictor of therapeutic outcomes across pharmacological and psychosocial modalities.

Sensory processing deficits represent a critical yet under-recognized dimension of schizophrenia, encompassing disturbances across visual, auditory, and somatosensory domains. These alterations likely reflect widespread abnormalities in neural networks and may contribute substantially to perceptual and cognitive dysfunction. A more precise understanding of their neurobiological underpinnings may inform the development of interventions targeting sensory processing mechanisms. Future research should prioritize mechanistic and longitudinal studies to clarify the temporal dynamics and clinical relevance of sensory abnormalities. In particular, further work is warranted to develop and evaluate interventions addressing specific sensory variables, including sensory anhedonia and tactile reactivity.

### Limitations

Our findings should be interpreted in light of several limitations. First, the sample of patients with schizophrenia was relatively small, which may limit generalizability. Second, the clinical and control groups differed in age and sex distribution. Although these imbalances reflect the clinical recruitment context and age and sex were statistically controlled, their potential influence cannot be fully excluded. Third, all patients were receiving pharmacological treatment, precluding evaluation of medication-free sensory responses. Antipsychotic dosing was not systematically recorded due to the dynamic nature of inpatient treatment. While participants were treated under standardized care conditions, medication effects on sensory perception remain a potential confound. Finally, to minimize acute pharmacological influences, participants were instructed to refrain from analgesic use on the day of assessment and were not administered benzodiazepines. Future studies involving larger, demographically matched samples and, where feasible, drug-naïve individuals are warranted.

## Conclusion

Individuals with schizophrenia exhibited altered sensory processing, as reflected in elevated thresholds for detecting electrical stimuli and a reduced tendency to report unpleasant sensations. This pattern may suggest altered sensory responsiveness, potentially involving reduced sensitivity to discomfort. Reduced hedonic responsiveness was significantly associated with increased age and longer treatment duration, indicating that the capacity for sensory pleasure may change across the course of illness. Moreover, anhedonia was associated with lower psychological and social quality of life, underscoring its functional and affective relevance. Taken together, these findings support the potential utility of integrating objective sensory assessments into the characterization of clinical phenotypes, particularly in relation to negative symptoms such as anhedonia. Sensory-based profiling may contribute to more individualized therapeutic and rehabilitative strategies aimed at improving adaptive functioning and emotional engagement in patients with schizophrenia.

## Supporting information

S1 Data Dataset 2023. (ZIP)
